# Ferroelectric Field‐Effect‐Transistor Integrated with Ferroelectrics Heterostructure

**DOI:** 10.1002/advs.202200566

**Published:** 2022-05-15

**Authors:** Sungpyo Baek, Hyun Ho Yoo, Jae Hyeok Ju, Panithan Sriboriboon, Prashant Singh, Jingjie Niu, Jin‐Hong Park, Changhwan Shin, Yunseok Kim, Sungjoo Lee

**Affiliations:** ^1^ SKKU Advanced Institute of Nanotechnology (SAINT) Sungkyunkwan University Suwon 440‐746 Korea; ^2^ School of Advanced Materials Science and Engineering Sungkyunkwan University (SKKU) Suwon 440‐746 Korea; ^3^ School of Electrical Engineering Korea University Seoul 02841 Korea; ^4^ Department of Nano Engineering Sungkyunkwan University Suwon 440‐746 Korea

**Keywords:** ferroelectric semiconductors, ferroelectronics, van der Waals ferroelectric heterostructures

## Abstract

To address the demands of emerging data‐centric computing applications, ferroelectric field‐effect transistors (Fe‐FETs) are considered the forefront of semiconductor electronics owing to their energy and area efficiency and merged logic–memory functionalities. Herein, the fabrication and application of an Fe‐FET, which is integrated with a van der Waals ferroelectrics heterostructure (CuInP_2_S_6_/*α*‐In_2_Se_3_), is reported. Leveraging enhanced polarization originating from the dipole coupling of CIPS and *α*‐In_2_Se_3_, the fabricated Fe‐FET exhibits a large memory window of 14.5 V at *V*_GS_ = ±10 V, reaching a memory window to sweep range of ≈72%. Piezoelectric force microscopy measurements confirm the enhanced polarization‐induced wider hysteresis loop of the double‐stacked ferroelectrics compared to single ferroelectric layers. The Landau–Khalatnikov theory is extended to analyze the ferroelectric characteristics of a ferroelectric heterostructure, providing detailed explanations of the hysteresis behaviors and enhanced memory window formation. The fabricated Fe‐FET shows nonvolatile memory characteristics, with a high on/off current ratio of over 10^6^, long retention time (>10^4^ s), and stable cyclic endurance (>10^4^ cycles). Furthermore, the applicability of the ferroelectrics heterostructure is investigated for artificial synapses and for hardware neural networks through training and inference simulation. These results provide a promising pathway for exploring low‐dimensional ferroelectronics.

## Graphical abstract

The authors report on the fabrication and application of a ferroelectric transistor integrated with a van der Waals ferroelectrics heterostructure (CuInP_2_S_6_/*α*‐In_2_Se_3_). Leveraging enhanced polarization originating from the dipole coupling, the fabricated device exhibits a large memory window and nonvolatile memory characteristics with long retention time and stable cyclic endurance, providing a promising pathway for exploring low‐dimensional ferroelectronics.

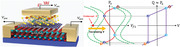

## 1 Introduction

Driven by the explosion of data‐centric applications, computing technology is evolving in an essentially different way that has been pursued with current complementary metal‐oxide‐semiconductor (CMOS) platforms. Fundamental innovations of electronic hardware in the computing hierarchy, down to the material and device levels, are required to deliver the demanded functionalities for emerging computing paradigms. Data‐centric computing devices require high performance, energy and area efficiency, and diverse merged logic–memory functionalities to support various workloads and applications. Ferroelectric field‐effect transistors (Fe‐FETs) have emerged as a forerunner of a new approach in electronic devices to address the future needs of computing. Conventionally, Fe‐FETs are viewed as three‐terminal nonvolatile devices containing a ferroelectric dielectric that controls electronic carriers in the underlying semiconductor in different polarization states induced by the gate electrode. It has been considered that Fe‐FETs could be dominant key components in future computing because of their attractive nondestructive readout functionality, low‐area footprint, and energy efficiency, along with their three‐terminal device structure. In particular, since the discovery of CMOS‐compatible HfO_2_‐based ferroelectric oxides,^[^ ^1^, ^2^ ^]^ many research activities have accelerated the development of Fe‐FETs, which can perform logic and memory functions in a single device.

However, conventional Fe‐FETs based on ferroelectric oxides face severe challenges for commercialization. For example, as reported in previous studies, in perovskite ferroelectrics containing BaTiO_3_ (BTO), Pb(Zr, Ti)O_3_ (PZT), and BiFeO_3_ (BFO), a small bandgap of 3–4 eV leads to leakage current and electrical breakdown,^[^ ^3^, ^4^ ^]^ and the perovskite ferroelectrics degrade during postannealing process, causing hindrances in the transistor fabrication process.^[^ ^5^ ^]^ Moreover, for CMOS‐compatible HfO_2_‐based ferroelectrics, the orthorhombic phase with ferroelectricity is induced only in a constrained thermal and chemical environment,^[^ ^6^ ^]^ and additional methods including postannealing processes (800–1000 °C),^[^ ^7^ ^]^ introduction of buffer layers such as TiN,^[^ ^8^ ^]^ and well‐controlled doping processes^[^ ^9^, ^10^ ^]^ are required to stabilize the ferroelectric properties. Additionally, the charge traps and voltage drops within the unavoidable low‐k interfacial oxide grown during the annealing process severely limit the memory retention time. Perhaps more crucially, the inherent operation mechanism of conventional Fe‐FETs, which indirectly controls the channel layer through the ferroelectric polarization of the gate dielectric, imposes fundamental limitations caused by inefficient channel controllability.

Recently, the advent of van der Waals (vdW) layered ferroelectrics such as CuInP_2_S_6_,^[^ ^11^ ^]^ BA_2_PbCl_4_,^[^ ^12^ ^]^ *α*‐In_2_Se_3_,^[^ ^13^ ^]^ GeS,^[^ ^14^ ^]^ SnS,^[^ ^15^ ^]^ and SnSe^[^ ^16^ ^]^ has provided a breakthrough for addressing the limitations of ferroelectric oxide‐based Fe‐FETs; therefore, significantly increasing the potential for the realization of multifunctional Fe‐FETs. In common, they exhibit strong ferroelectricity down to a few atomic layers at room temperature. In addition, their dangling bond‐free surface, an intrinsic property of vdW materials, provides a solution to overcome charge trapping and interfacial oxide issues at the ferroelectric/semiconductor interface and enable the formation of high‐quality van der Waals heterostructures (vdWHs) without lattice mismatch issues. Although the existence of vdW ferroelectricity was predicted in 1976,^[^ ^17^ ^]^ experimental demonstrations were only reported very recently. Belianinov et al.^[^ ^18^ ^]^ reported ferroelectric CuInP_2_S_6_ (CIPS) down to 50 nm experimentally, as evidenced by domain structures, rewritable polarization, and hysteresis loops. Susner et al.^[^ ^19^ ^]^ showed enhanced ferroelectric properties in CIPS as thin as 20 nm. In 2018, Si et al.^[^ ^20^ ^]^ reported a vdWH Fe‐FET integrated with a MoS_2_ channel and CIPS. Their study showed the potential for nonvolatile memory utilizing vdW ferroelectrics, but the fundamental limitation of inefficient channel controllability still remains owing to the use of ferroelectric dielectrics. More recently, ferroelectric semiconductor field‐effect transistors (FeS‐FETs) using the ferroelectric semiconductor *α*‐In_2_Se_3_ as a channel layer were reported.^[^ ^13^, ^21^, ^22^ ^]^ Two nonvolatile polarization states are formed in the channel layers, and they showed that the limitations of conventional Fe‐FETs, such as charge trap and leakage current problems, can be overcome by directly controlling the ferroelectric polarization in the channel layers. Although several studies have been conducted using various vdW ferroelectrics,^[^ ^13^, ^14^, ^15^, ^16^, ^23^, ^24^, ^25^ ^]^ the cointegration of *α*‐In_2_Se_3_ and CIPS and their consequent device performances have never been reported.

Herein, we report an all‐ferroelectric (i.e., ferroelectric dielectric + ferroelectric semiconductor) field‐effect transistor (Fe‐FET) utilizing ferroelectric materials in the gate dielectric and channel layers, which are the active components of the FET structure. Our Fe‐FET device was composed of vdW‐layered materials. Specifically, CIPS, h‐BN, and *α*‐In_2_Se_3_ flakes were used as the ferroelectric gate dielectric, insulating, and ferroelectric semiconducting channel layers, respectively. The fabricated Fe‐FET device demonstrates a large memory window of 14.47 V at a voltage sweep of ±10 V, a high on/off current ratio of higher than 10^6^, stable long retention time (>10^4^ s), and stable cyclic endurance (>10^4^ cycles) at room temperature. These superior performance metrics can be attributed to dipole coupling occurring at the *α*‐In_2_Se_3_/CIPS interface. Furthermore, we extended the Landau–Khalatnikov (L–K) theory, which considers only the relationship between the ferroelectric and dielectric, to the relationship between two ferroelectrics. Our theoretical analysis explains the enhanced ferroelectric properties of the integrated ferroelectric‐dielectric/ferroelectric‐semiconductor structure, evidencing a wider hysteresis window. Furthermore, we demonstrate the applicability of our ferroelectrics heterostructure device for artificial synapses by mimicking the dynamics of biological synapses, such as excitatory/inhibitory postsynaptic currents (EPSC/IPSC), paired‐pulse facilitation (PPF), and long‐term potentiation/depression (LTP/LTD) characteristics. Moreover, the applicability for hardware neural networks is demonstrated through training and inference simulation using a convolutional neural network (CNN) and Canadian‐Institute‐For‐Advanced‐Research‐10 (CIFAR‐10) datasets.^[^ ^26^, ^27^ ^]^

## 2 Results and Discussion

**Figure 1:**
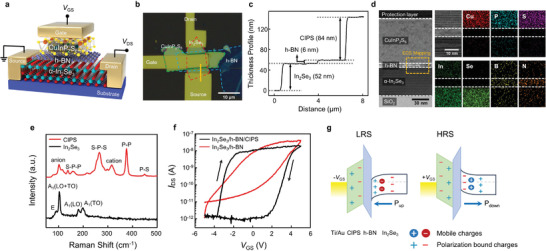
a) Schematic of three‐terminal vdWH Fe‐FET device fabricated with CuInP_2_S_6_, h‐BN, and *α*‐In_2_Se_3_. b) Optical image of vdWH Fe‐FET device based on *α*‐In_2_Se_3_/h‐BN/CIPS heterostructure. c) Thickness profile of *α*‐In_2_Se_3_ (52 nm)/h‐BN (6 nm)/CIPS (84 nm) heterostructure. The thickness was measured along the gold line in b). d) Cross‐sectional TEM image and corresponding EDS element mapping of *α*‐In_2_Se_3_/h‐BN/CIPS heterostructure. e) Raman spectra for *α*‐In_2_Se_3_ and CIPS. f) Transfer characteristics of *α*‐In_2_Se_3_/h‐BN/CIPS (black) and *α*‐In_2_Se_3_/h‐BN (red) devices at room temperature. The voltage, *V*_GS_, was swept from −5 to +5 V, and the drain current was measured at the voltage of *V*_DS_ = 1 V. g) Energy band diagrams of *α*‐In_2_Se_3_/h‐BN/CIPS Fe‐FET device at P_up_ state (left) and P_down_ state (right).

**Figure** **1**a shows the schematic of a vdWH Fe‐FET integrated with CIPS, h‐BN, and *α*‐In_2_Se_3_, where CIPS, h‐BN, and *α*‐In_2_Se_3_ act as the ferroelectric gate dielectric, insulating, and ferroelectric semiconducting channel layers, respectively. Each flake was prepared by the mechanical exfoliation method and then sequentially transferred onto the SiO_2_/Si substrate via the dry transfer method.^[^ ^28^ ^]^ Layers of Ti (10 nm) and Au (80 nm) were deposited for the source, drain, and top gate electrodes. The detailed fabrication processes are provided in the Experimental Section. An optical image of the fabricated device is shown in Figure 1b, where the red, blue, and green dashed lines represent *α*‐In_2_Se_3_, h‐BN, and CIPS, respectively. The layer thicknesses are 52 nm (*α*‐In_2_Se_3_), 6 nm (h‐BN), and 84 nm (CIPS), as shown in Figure 1c. The atomic force microscopy (AFM) topography image of Figure 1c is provided in Figure S1a (Supporting Information). Figure 1d shows the cross‐sectional transmission electron microscopy (TEM) image and corresponding energy dispersive X‐ray spectroscopy (EDS) element mapping of the *α*‐In_2_Se_3_/h‐BN/CIPS heterostructure of Figure 1b. The vdW heterostructure with a charge trap‐free interface can be implemented without lattice mismatch, owing to the dangling bond‐free surface nature.^[^ ^29^, ^30^ ^]^ The corresponding Raman peaks for each layer also indicate the existence of *α*‐In_2_Se_3_, CIPS (Figure 1e), and h‐BN (Figure S1, Supporting Information) in the fabricated vdWHs.

Figure 1f shows the transfer characteristics of both ferroelectric FET devices: FeS‐FET (*α*‐In_2_Se_3_/h‐BN) and vdWH Fe‐FET (*α*‐In_2_Se_3_/h‐BN/CIPS). The detailed device structure and dimensions of the *α*‐In_2_Se_3_/h‐BN FeS‐FET are shown in Figure S2 (Supporting Information). While both devices show n‐type behavior and clockwise hysteresis, compared to FeS‐FET, a greatly enhanced memory window was obtained from the vdWH Fe‐FET. This result indicates that the ferroelectric polarization of CIPS enhances the conduction behavior of the ferroelectric *α*‐In_2_Se_3_ channel. The operating principle can be understood through the energy band diagrams of the *α*‐In_2_Se_3_/h‐BN/CIPS structure in polarization up and polarization down states (Figure 1g). Unlike ferroelectric dielectrics (CIPS), which have only polarization‐bound charges, ferroelectric semiconductors (*α*‐In_2_Se_3_) have mobile and polarization‐bound charges because their semiconducting and ferroelectric natures are coupled. We note that the applied electric field cannot fully penetrate the channel layer owing to the relatively high thickness of the gate insulator layer, resulting in partial ferroelectric switching and localized mobile charge distribution. When a negative (positive) gate bias below (above) the coercive voltage (*V*_c_) is applied, the polarization‐bound charges in the CIPS and *α*‐In_2_Se_3_ layers are arranged in a polarization up (down) state. Consequently, downward (upward) band bending and accumulation (depletion) of the channel occur at the top surface of *α*‐In_2_Se_3_ to form a low (high) resistance state. This operating principle leads to clockwise hysteresis. Owing to the presence of the polarization‐bound charges in the gate dielectric as well as in the channel layer, when the dipoles of the CIPS and *α*‐In_2_Se_3_ are arranged in one direction by an external electric field, they are coupled to each other, enabling the polarization states to be maintained for a long time and the achievement of a larger memory window.

The ferroelectric characteristics of different layer‐stacked structures, including Au/*α*‐In_2_Se_3_/h‐BN/CIPS/PFM‐tip structures, were measured by piezoelectric force microscopy (PFM).^[^ ^31^ ^]^

**Figure 2:**
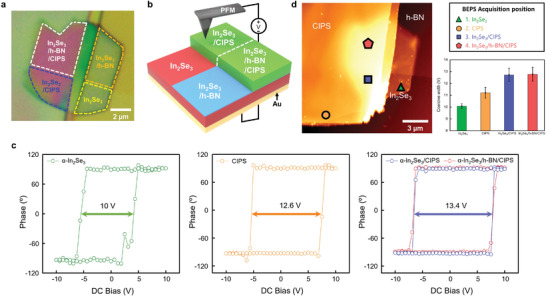
a) OM image of Au/*α*‐In_2_Se_3_/h‐BN/CIPS/PFM‐tip structure. The yellow, orange, blue, and white dotted boxes indicate *α*‐In_2_Se_3_, *α*‐In_2_Se_3_/h‐BN, *α*‐In_2_Se_3_/CIPS, and *α*‐In_2_Se_3_/h‐BN/CIPS structures, respectively. b) Schematic of the structure is shown in a). c) PFM phase hysteresis of *α*‐In_2_Se_3_ (green), CIPS (orange), *α*‐In_2_Se_3_/CIPS (blue), and *α*‐In_2_Se_3_/h‐BN/CIPS (red) structures. Each hysteresis loop represents the average of nine measurements. d) AFM topography image of Au/*α*‐In_2_Se_3_/h‐BN/CIPS/AFM‐tip structure (left), acquisition position of PFM hysteresis loops (upper right), and coercive width of each position (lower right). The coercive width is defined as an absolute difference between negative and positive coercive voltages.

**Figure 3:**
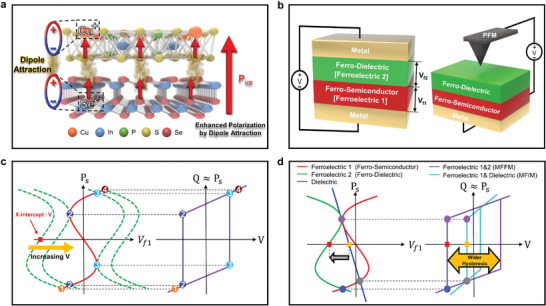
a) Schematic atomic structure illustrating polarization up state of *α*‐In_2_Se_3_/CIPS structure. b) Schematic of MFFM Capacitor (left) and PFM measurement (right). c) Process of creating hysteresis window. The green s‐curve shifts as *V* increases and the two s‐curves intersect each other (left). When connecting those intersections, hysteresis is formed (right). d) Comparison of hysteresis window for ferroelectric/ferroelectric and ferroelectric/dielectric structures.

**Figure** **2**a,b shows an optical microscopy (OM) image and schematic of the measurement setup of the PFM, respectively. The fabrication process and thickness profiles of the PFM sample structures are shown in Figure S3 (Supporting Information). Figure 2c shows the PFM phase hysteresis loops for *α*‐In_2_Se_3_ (green), CIPS (orange), and *α*‐In_2_Se_3_/CIPS (blue). Compared with *α*‐In_2_Se_3_ (10 V) and CIPS (12.6 V), a wider coercive width (13.4 V) from the *α*‐In_2_Se_3_/CIPS vdWH is observed owing to the dipole attraction of the polarized charges between the two ferroelectrics. Detailed descriptions on obtaining hysteresis enhancement of the double‐stacked ferroelectrics are presented in **Figure** **3**. The *α*‐In_2_Se_3_/h‐BN/CIPS (red) structure, which was used for Fe‐FET fabrication to reduce the leakage current, showed a PFM phase hysteresis loop similar to that of the *α*‐In_2_Se_3_/CIPS structure. The left side of Figure 2d shows the AFM topography image, and each shape represents the acquisition positions. As shown on the upper right side of Figure 2d, the triangle represents *α*‐In_2_Se_3_, the circle represents CIPS, and the square represents *α*‐In_2_Se_3_/CIPS, the pentagon represents *α*‐In_2_Se_3_/h‐BN/CIPS, respectively. To verify the uniformity of the coercive width, each coercive width with an error bar is shown in Figure 2d (bottom right). In addition, the PFM phase, amplitude, and piezoresponse hysteresis loop of each structure are provided in Figure S4 (Supporting Information).

The ferroelectric characteristics of the ferroelectrics heterostructure (i.e., the *α*‐In_2_Se_3_/CIPS structure) and the consequent hysteresis behaviors are explained in Figure 3. Figure 3a shows a schematic of the interaction between *α*‐In_2_Se_3_ and CIPS on an atomic scale. When these two ferroelectric materials are polarized in the upward direction, Cu^+^ ions in CIPS move upward, and Se^2−^ ions in *α*‐In_2_Se_3_ move downward, forming dipoles.^[^ ^32^, ^33^ ^]^ In this double‐stacked ferroelectrics, the overall polarization will be enhanced with dipole attraction between two ferroelectric materials, creating a wider hysteresis window and longer polarization, as shown in Figure 3c,d. A metal/ferroelectric 1/ferroelectric 2/metal (MFFM) capacitor (Figure 3b) was used to investigate the hysteresis of the ferroelectric heterostructure. Based on the L–K theory,^[^ ^34^ ^]^ each ferroelectric can have two stable polarization states, and the total Gibbs free energy per area of this MFFM system can be written as

(1) $$\begin{array}{c} G_{\mathrm{tot}}=G_{f1}t_{f1}+G_{f2}t_{f2}=\left(\alpha t_{f1}+xt_{f2}\right)P_{s}^{2}+\left(\beta t_{f1}+yt_{f2}\right)P_{s}^{4}-VP_{s} \end{array}$$

*G*_f1_ is the Gibbs free energy of the ferroelectric semiconductor, and (*α*, *β*) is the Landau coefficient of *G*_f1_. *G*_f2_ is the Gibbs free energy of the ferroelectric dielectric, and (*x*, *y*) is the Landau coefficient of *G*_f2_. The thicknesses of the ferroelectrics are *t*_f1_ and *t*_f2_. *P_s_* is the spontaneous polarization. *V* is the voltage of the MFFM capacitor (*V*  = *V*_f1_  + *V*_f2_). From Equation (1), *G*_tot_ can have two stable polarization states because *G*_tot_ has a W‐shaped curve, as shown in Figure S5a (Supporting Information). Therefore, the point where *P*_s_ can exist is where the derivative of *G*_tot_ with respect to *P*_s_ becomes 0. By differentiating and modifying Equation (1), it can be expressed as

(2) $$V_{f1}=V-2xt_{f2}P_{s}-4yt_{f2}P_{s}^{3}$$

Equation (2) is an expression for the variables *V*_f1_ and *P*_s_, which reveals how the polarization is affected when the voltage changes in the MFFM capacitor. Detailed explanations on the basis and extension process of the L–K theory for ferroelectric heterostructures can be found in Figures S5–S8 (Supporting Information). Figure 3c visualizes Equation (2) as a graph with *V*_f1_ and *P_s_* axes. In Figure 3c, the red S‐curve represents the left side of Equation (2), and the green S‐curve represents the right side of Equation (2). A detailed explanation of the red and green S‐curves is provided in Figure S6 (Supporting Information). Figure 3c shows the formation process of hysteresis when a voltage is applied to the MFFM capacitor. On the left side of Figure 3c, the intersections represent stable polarization states in the ferroelectric heterostructure. Initially, the green and red curves only have one intersection corresponding to 1. As the voltage applied to the MFFM capacitor increases, the applied voltage *V* corresponding to the red square represents the x‐intercept of the green curve, and the green curve shifts to the red curve along the *V*_f1_ axis. Subsequently, the green and red curves have two intersections at 2 and 3, respectively. The two intersections at the red and green curves indicate that the total system can have two distinct stable polarizations. As the voltage further increases, the two curves intersect at only one point, corresponding to 4. The right side of Figure 3c shows the *Q* − −*V* curve of the ferroelectric heterostructure. *Q* is the charge of the entire system, which can be approximated as *Q* ≈ *P*_s_. Therefore, the intersections between the red and green curves can be the points of the *Q* − −*V* curve. Consequently, a hysteresis loop was formed when connecting the points of the *Q* − −*V* curve, as shown in Figure 3c. Figure 3d compares the hysteresis behaviors of the MFFM and metal/ferroelectric/insulator/metal (MFIM) structures. Details about the L–K theory extension and hysteresis formation behavior of the MFIM capacitor are provided in Figures S7 and S8 (Supporting Information). In Figure 3d, *P*_s_ − −*V*_f1_ curve (left) and *Q* − −*V* curve (right) of the MFFM (red/green in left and violet in right) are compared with those of the MFIM (red/blue in left and cyan in right), which is composed of ferroelectric 1 and a nonferroelectric dielectric. It can be observed that the MFFM (ferroelectric 1 and 2) has a wider hysteresis, which is determined by the x‐intercepts, owing to different slopes caused by different energy landscape natures. This theoretical analysis was experimentally confirmed from the PFM‐measured hysteresis window comparison of these two structures (*α*‐In_2_Se_3_/h‐BN and *α*‐In_2_Se_3_/CIPS) fabricated on the same *α*‐In_2_Se_3_ film (Figure S9, Supporting Information).

**Figure 4:**
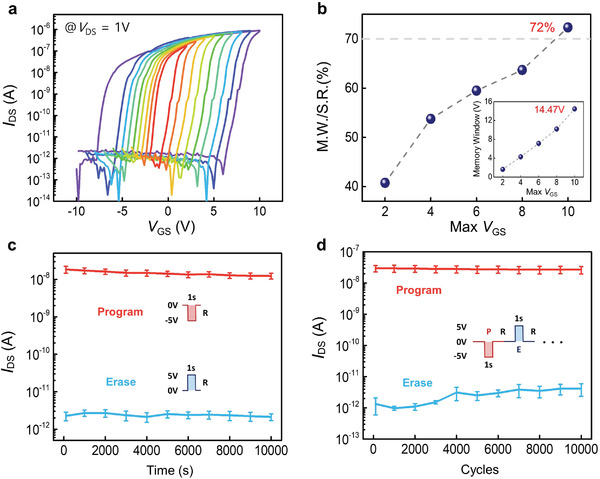
a) Transfer characteristics of *α*‐In_2_Se_3_/h‐BN/CIPS Fe‐FET device under different *V*_GS_ sweep range. b) M.W./S.R ratios as a function of *V*_GS_ sweep range. The ratio value reached ≈72% when the max *V*_GS_ was 10 V. Inset shows memory windows as a function of max *V*_GS_. The memory window was linearly increased as the *V*_GS_ sweep ranges increased. The largest memory window of 14.47 V was obtained when the max *V*_GS_ was 10 V. c) Data retention characteristic for 10^4^ s after applying programing/erasing pulses. d) Endurance characteristic upon 10^4^ cycles of programing/erasing pulses.

Next, we investigated the room‐temperature memory characteristics of the vdWH Fe‐FET device based on the *α*‐In_2_Se_3_/h‐BN/CIPS structure_._ **Figure** **4**a shows the transfer characteristics controlled by the top gate. The sweep range of *V*_GS_ increased from ±2 to ±10 V, and the drain current was measured at *V*_DS_ = 1 V. The measured transfer curves exhibit n‐type behavior and clockwise hysteresis with a high on/off current ratio of over 10^6^ and a large memory window of 14.47 V under *V*_GS_ = ±10 V. This result can be attributed to ferroelectric polarization switching and coupled dipoles of CIPS and *α*‐In_2_Se_3_, as explained above. Figure 4b shows the memory window and the ratio of the memory window (M.W.) to sweep range (S.R.) with respect to the *V*_GS_ sweep range. The memory window linearly increased as the *V*_GS_ sweep ranges increased, showing the memory window of 14.47 V and the M.W./S.R. ratio value of 72% at max *V*_GS_ = 10 V. The M.W./S.R. ratio is needed for the performance comparison because all ferroelectrics used in ferroelectric FETs reported previously have different material properties according to the device structure and thickness. Table S1 (Supporting Information) shows that the M.W./S.R. ratio value of the *α*‐In_2_Se_3_/h‐BN/CIPS Fe‐FET is superior to those of the ferroelectric memory devices reported thus far. Furthermore, the fabricated Fe‐FET exhibited insignificant hysteresis window changes under different drain biases, and no sign of degradation was detected after multiple *V*_GS_ sweeps (Figure S10, Supporting Information). Data retention characteristics were investigated by applying programming/erase pulses with an amplitude of ±5 V and a duration of 1 s at a voltage, *V*_DS_, of 1 V (Figure 4c). The drain current was measured after applying the programming/erasing pulses, and highly stable program/erase states without significant degradation could be maintained for longer than 10^4^ s with a P/E current ratio of 10^4^. Figure 4d shows the endurance characteristics as a function of the number of cycles, demonstrating two stable states for 10^4^ cycles. Figure 4c,d shows the average results of the four *α*‐In_2_Se_3_/h‐BN/CIPS Fe‐FET devices. The relevant raw data are provided in Figure S11 (Supporting Information).

**Figure 5:**
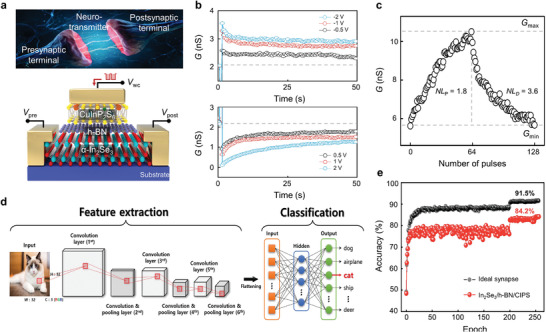
a) Structural comparison of biological synapse and three‐terminal vdWH Fe‐FET synaptic device. b) Excitatory (top) and inhibitory (bottom) postsynaptic conductance responses (EPSC/IPSC) with respect to the amplitude of the electrical pulse applied to weight control terminal (WCT). c) LTP/LTD curve with *NL* of 1.8/3.6, where 64/64 excitatory/inhibitory pulses with an amplitude of ±0.5 V, a duration of 5 ms, and a frequency of 2 Hz were applied. d) Schematic of convolutional neural network, which consists of six convolutional layers and two fully connected layers. e) Image recognition accuracy as a function of epoch. The black and red lines are the results for the ideal device and vdWH Fe‐FET device, respectively.

The synaptic characteristics were investigated to evaluate the applicability of the vdWH Fe‐FET device for artificial synapses. **Figure** **5**a shows a schematic of this synaptic device, which mimics the dynamics of a biological synapse. The channel conductivity related to the synaptic weight is potentiated or depressed owing to partial ferroelectric switching caused by applying electrical stimulation at the weight control terminal (WCT). Figure 5b shows the postsynaptic current (PSC) responses caused by excitatory and inhibitory *V*_wc_ pulses. *V*_wc_ pulses with an amplitude ranging from ±0.5 to ±2 V and a duration of 100 ms were applied to the WCT for EPSC and IPSC characteristics, and the conductance (*G* = *I*_psc_/*V*_post_) was measured by applying *V*_post_ of 1 V. Under varied *V*_wc_ pulses, the conductance increased by 12.2%, 34.8%, and 41.9% from the initial state for EPSC, whereas decreased by 20.0%, 32.0%, and 41.5% for IPSC. In both cases, after *V*_wc_ pulses were applied, the conductance did not return to the initial state for a duration of 50 s. Moreover, the PPF characteristics related to short‐term plasticity were investigated, and the details are provided in Figure S12 (Supporting Information). Subsequently, the LTP/LTD characteristics related to long‐term plasticity were examined by applying 64/64 excitatory/inhibitory *V*_wc_ pulses (amplitude of ±0.5 V, duration of 5 ms, and frequency of 2 Hz), as shown in Figure 5c. When 64 excitatory *V*_wc_ pulses were consecutively applied to the WCT, the conductance level potentiated from 10.0 to 22.6 nS with the nonlinearity (*NL*) of 1.8. By contrast, when 64 inhibitory *V*_wc_ pulses were applied, the conductance level depressed 22.6–9.31 nS with the *NL* of 3.6. When a series of electrical pulses are applied, the dipoles of the CIPS and *α*‐In_2_Se_3_ are gradually switched by the induced E‐field, resulting in a gradual conductance change. The *NL* extraction method and LTP/LTD curves investigated under various pulse conditions are shown in Figures S13 and S14 (Supporting Information).^[^ ^35^, ^36^, ^37^ ^]^ Finally, we demonstrate its applicability to hardware neural networks (HW‐NNs) using the “DNN+ NeuroSim” simulator.^[^ ^38^ ^]^ The artificial neural network of the simulator was a CNN, and the Canadian Institute for Advanced Research‐10 (CIFAR‐10) image dataset was used for training (50 000 images) and inference (10 000 images) tasks.^[^ ^26^, ^27^ ^]^ A schematic of the CNN, which consists of six convolutional layers for feature extraction and two fully connected layers for classification, is shown in Figure 5d. HW‐NN used in this work consists of *α*‐In_2_Se_3_/h‐BN/CIPS ferroelectric synaptic devices for the fully connected CNN layer, where the synaptic weight (W) is defined as the difference between the conductance values of two equivalent ferroelectric synapses, W = *G*_P_ – *G*_D,_ where *G*_P_ and *G*_D_ denote the conductance values for potentiating and depressing, respectively. As a result, the image recognition accuracy of the Fe‐FET, estimated from 50 000 training and 10 000 inference tasks per epoch, reached 84.2%. The detailed information on the network configuration, image data processing, and the relationship between NNs and synaptic characteristics are provided in Figure S17 (Supporting Information).

## 3 Conclusion

In this study, we report a vdWH Fe‐FET device fabricated with a ferroelectric dielectric (CIPS) and a ferroelectric semiconductor (*α*‐In_2_Se_3_). Since the dipole coupling that occurs at the *α*‐In_2_Se_3_/CIPS interface leads to longer polarization, the *α*‐In_2_Se_3_/h‐BN/CIPS Fe‐FET device shows a large memory window of 14.47 V under *V*_GS_ = ±10 V. The results of PFM also show that the ferroelectric heterostructure has a large hysteresis characteristic compared to other structures. In addition to the experimental analyses, the reasons for the hysteresis expansion when integrating two ferroelectrics were analyzed through the extended L–K theory, which considers the relationship between ferroelectric dielectrics and ferroelectric semiconductors. In addition to a large memory window, a high on/off current ratio of over 10^6^, long retention time, and stable cyclic endurance were obtained at room temperature. Finally, we implemented an artificial vdWH Fe‐FET synapse device, which successfully mimicked the dynamics of biological synapses such as EPSC/IPSC, PPF, and LTP/LTD characteristics. Additionally, we demonstrated the applicability of the presented device to hardware neural networks through training and inference simulations using CNN and CIFAR‐10 datasets. These results highlight the promise of the vdWH Fe‐FET as a forerunner of low‐dimensional ferroelectricity in addressing the future needs of data‐centric computing applications.

## 4 Experimental Section

### Device Fabrication

*α*‐In_2_Se_3_, h‐BN, and CIPS flakes (HQ graphene) were mechanically exfoliated on polydimethylsiloxane (PDMS) films and sequentially transferred onto a cleaned SiO_2_/Si substrate using the dry transfer method. The electrodes were patterned by electron beam lithography on the *α*‐In_2_Se_3_/h‐BN/CIPS heterostructure, and then metal layers of Ti (10 nm) and Au (80 nm) were deposited using an electron beam evaporator.

### Characterization

An optical microscope (Olympus, BX51M) was used to confirm the size and shape of the flakes and devices. The thickness of the flakes was measured by AFM (Park Systems, NX‐10) in the noncontact mode. A cantilever (Multi75E‐G, Park Systems, nominal spring constant k ≈ 3 N m^−1^) with a Cr 5 nm/Pt 25 nm coating on both sides was employed to obtain an AFM topography image. AFM scanning was conducted at a set point of 70 nN and at a scan rate of 0.8 Hz. Band excitation (BE)‐PFM measurements were performed using an AFM system that was equipped with Cr/Pt‐coated conductive tips (Multi75E‐G, BudgetSensors, nominal spring constant k ≈ 3 N m^−1^), a function generator, and a data acquisition system (NI‐PXIe 5122/5412, National Instruments) with LabVIEW/MATLAB software.^[^ ^39^, ^40^ ^]^ The frequency bandwidth and AC amplitude of the BE measurements were 80 kHz and 1 V, respectively. Raman spectroscopy was performed using a laser with an excitation wavelength of 532 nm (Kaiser Optical Systems, Model RXN). Cross‐sectional TEM (JEOL, JEM ARM 200F) measurements were conducted for the structural analysis. The electrical characteristics were investigated at room temperature using a Keysight B2912A instrument.

## Conflict of Interest

The authors declare no conflict of interest.

## Supporting information

Supporting Information

## Data Availability

The data that support the findings of this study are available from the corresponding author upon reasonable request.
