# Supplementary material for: Ferroelectric Field‐Effect‐Transistor Integrated with Ferroelectrics Heterostructure
Source: Adv Sci (Weinh). 2022 May 15;9(21):2200566. doi: 10.1002/advs.202200566 (PMC9313508; doi:10.1002/advs.202200566)
Supplement: Supplementary file 1 — Supporting Information [file ADVS-9-2200566-s001.pdf]

ADVANCED SCIENCE
Open Access

Supporting Information

for *Adv. Sci.*, DOI 10.1002/advs.202200566

Ferroelectric Field-Effect-Transistor Integrated with Ferroelectrics Heterostructure

*Sungpyo Baek, Hyun Ho Yoo, Jae Hyeok Ju, Panithan Sriboriboon, Prashant Singh, Jingjie Niu, Jin-Hong Park, Changhwan Shin, Yunseok Kim and Sungjoo Lee**

Supporting Information

AFM topography of vdWH Fe-FET and Raman spectrum of h-BN

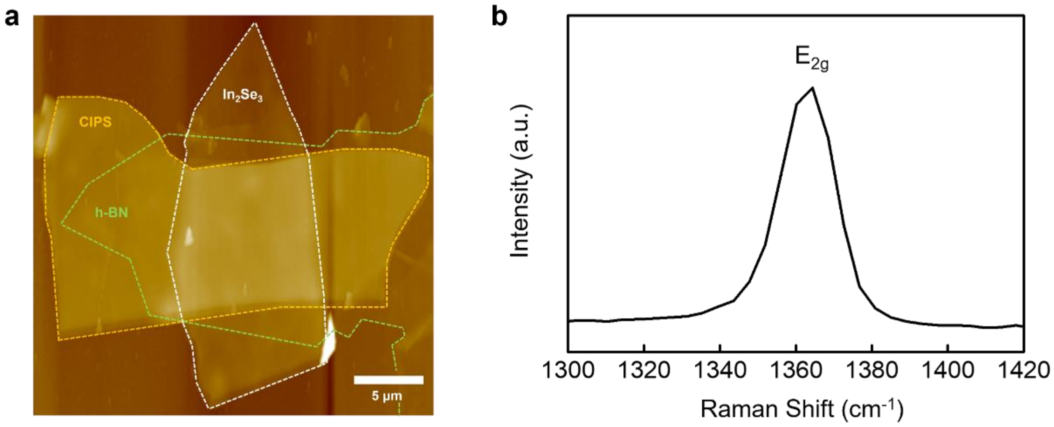

Figure S1. a) AFM topography of vdWH Fe-FET device based on α-In_2Se_3/h-BN/CIPS heterostructure. b) Raman spectrum corresponding to h-BN in α-In_2Se_3/h-BN/CIPS heterostructure.

Figure S1a shows AFM topography image of Figure 1c. Figure S1b shows the Raman spectrum corresponding to the h-BN layer in the α-In_2Se_3/h-BN/CIPS heterostructure. The Raman peak for the E_{2g} phonon mode of the h-BN flake was observed at 1365 cm^{-1}, which is consistent with the results of previous reports.[S1]

Characterization of FeS-FETs fabricated with α-In_2Se_3/h-BN heterostructures

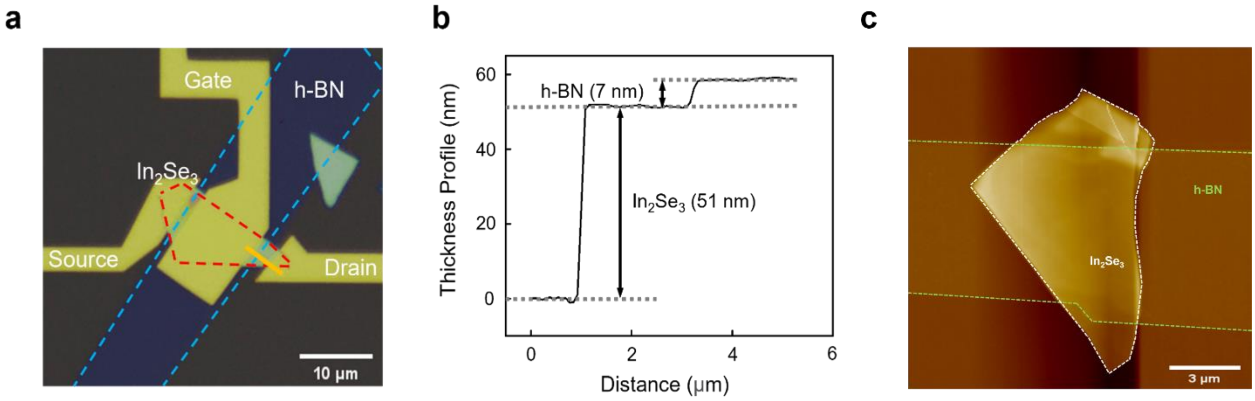

Figure S2. a) Optical image of FeS-FET device based on α-In_2Se_3/h-BN heterostructure. b) Thickness profile of α-In_2Se_3/h-BN heterostructure (along gold line in Figure S2a). c) AFM topography image of FeS-FET device based on α-In_2Se_3/h-BN heterostructure.

Figure S2a shows an optical image of the FeS-FET device based on the α-In_2Se_3/h-BN heterostructure. The red and blue dashed lines indicate α-In_2Se_3 and h-BN, respectively. Figure S2b shows the thickness profile of the α-In_2Se_3 (51 nm)/h-BN (7 nm) heterostructure measured along the gold line shown in Figure S2a. AFM topography image is shown in Figure S2c.

Fabrication process and thickness profiles of the PFM sample structures

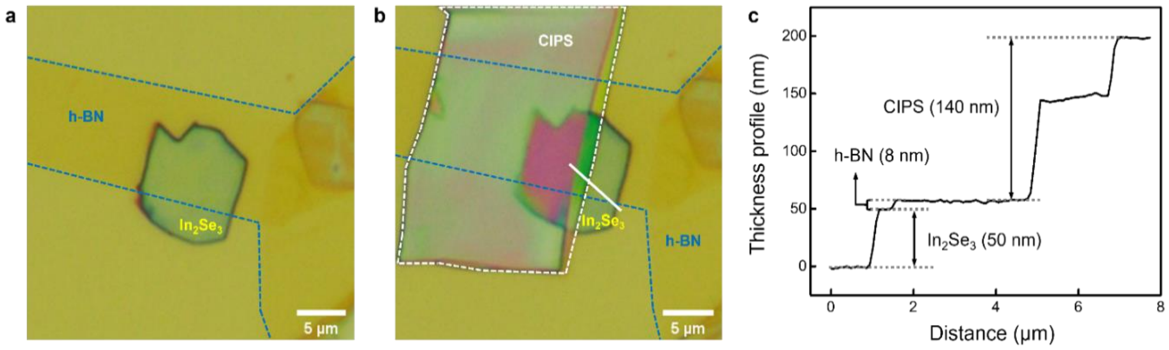

Figure S3. a) OM image of α-In_2Se_3/h-BN structure. b) OM image of α-In_2Se_3/h-BN/CIPS structure. c) Thickness profile of metal/ferroelectric/insulator/ferroelectric/metal (along white line Figure S3b).

"Figure S3a and b shows the fabrication process of the MFIFM structure. Initially, a 50-nm α-In_2Se_3 flake was stacked on the Au substrate. Subsequently, half of the α-In_2Se_3 flake was covered with an 8-nm h-BN layer horizontally (Figure S3a). Finally, the other half of the α-In_2Se_3 flake (Figure S3b) was covered with a 140-nm CIPS vertically. Figure S3c shows the thickness profile of the α-In_2Se_3 (50 nm)/h-BN (8 nm)/CIPS (140 nm) structure."

PFM phase, amplitude, and piezoresponse hysteresis loop of each structure

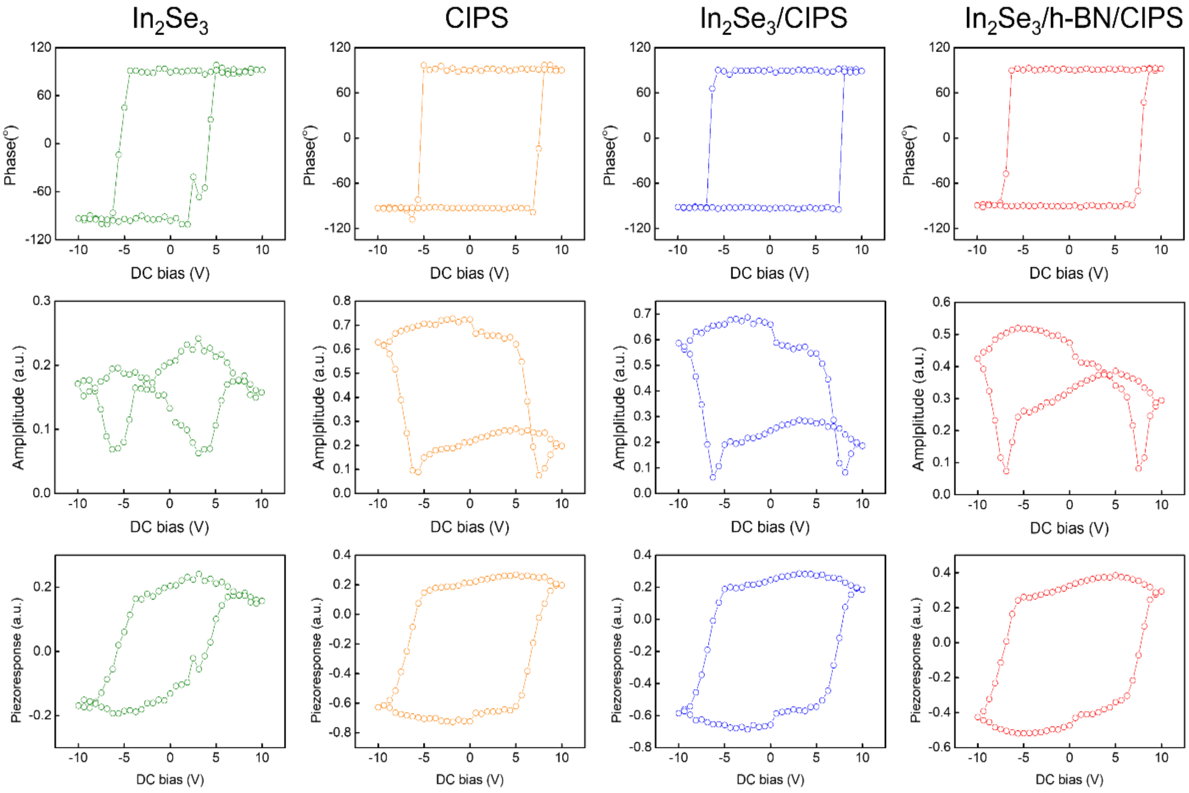

Figure S4. PFM phase, amplitude, and piezoresponse hysteresis loop of each structure. Each hysteresis loop represents the average of nine measurements.

Landau–Khalatnikov theory to explain time dynamics of ferroelectric switching

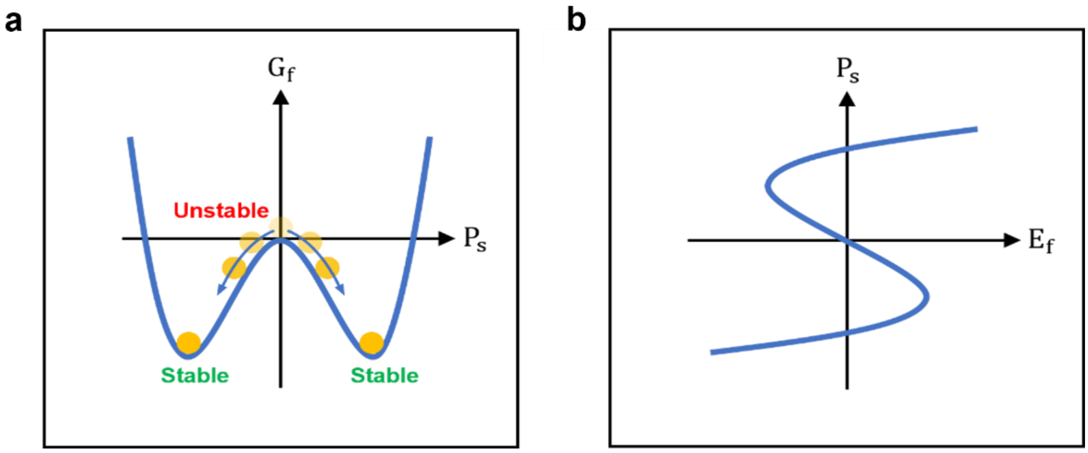

Figure S5. a) Energy landscape of ferroelectric material. The two minima reveal stable spontaneous polarization states. b) Ferroelectric S-shape curve with axes P_s (polarization) and E_f (electric field).

Ferroelectric materials can have two different polarization states, even though the E-field is not induced because of its noncentrosymmetric structure.[S2] Based on these characteristics, the energy landscape of ferroelectrics can be modeled using the Landau–Khalatnikov (L–K) equation.[S3] Therefore, the Gibbs free energy of the ferroelectric is expressed as

$$G_f = \alpha P_s^2 + \beta P_s^4 - E_f P_s \quad \text{(S1)}$$

In Eq.(S1), E_f is the electric field and P_s is the spontaneous polarization. In Figure S5a, the W-shaped curve has two minimum points, which means that it can have two stable spontaneous polarization states. Note that because ferroelectrics change to a paraelectric above the Curie temperature, we consider the ferroelectrics to be lower than the Curie temperature. Moreover, the Landau coefficients corresponding to $(\alpha < 0)$ and $(\beta > 0)$ are assumed to be second-order phase transitions.[S4] Figure S5b shows the S-curve, revealing the expression where the derivative of G_f with respect to P_s becomes 0. Thus, the expression of the S curve can be written as

$$E_f = 2\alpha P_s + 4\beta P_s^3 \quad \text{(S2)}$$

L–K theory extension for MFFM capacitor

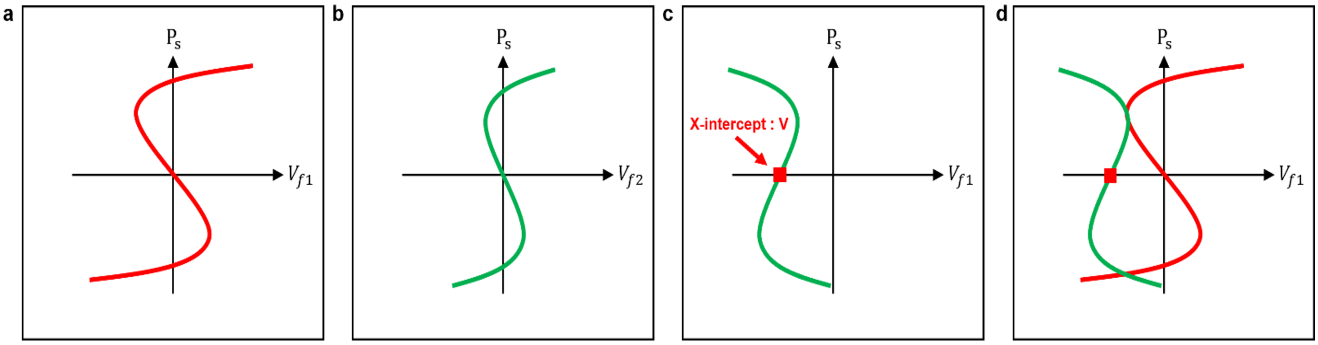

Figure S6. a) Ferroelectric S-curve of Eq. (S8). b) Ferroelectric S-curve of Eq. (S9). c) Curve with negative slope of figure S6b, and x-intercept represent *V* corresponding to the red square. d) Curves in S6a and Figure S6c are together in one quadrant.

Based on the L-K equation, the Gibbs free energy of each ferroelectric in a metal/ferroelectric semiconductor/ferroelectric dielectric/metal (MFFM) capacitor is given by

$$G_{f1} = \alpha P_s^2 + \beta P_s^4 - E_{f1}P_s \text{ (S3)},\ G_{f2} = xP_s^2 + yP_s^4 - E_{f2}P_s \text{ (S4)}$$

(α, β) is the Landau coefficient of G_{f1} and (x, y) is the Landau coefficient of G_{f2}.[S4]
Therefore, the total free energy of the MFFM capacitor per area can be written as

$$G_{tot} = G_{f1}t_{f1} + G_{f2}t_{f2} = (\alpha t_{f1} + xt_{f2})P_s^2 + (\beta t_{f1} + yt_{f2})P_s^4 - E_{f1}t_{f1}P_s - E_{f2}t_{f2}P_s \text{ (S5)}$$

As mentioned above, because $(V_{f1} = E_{f1}t_{f1}, V_{f2} = E_{f2}t_{f2})$, G_{tot} can be modified as

$$G_{tot} = G_{f1}t_{f1} + G_{f2}t_{f2} = (\alpha t_{f1} + xt_{f2})P_s^2 + (\beta t_{f1} + yt_{f2})P_s^4 - VP_s \text{ (S6)}$$

Because Eq. (S6) is in the same form as Eq. (S3, S4), G_{tot} is a W-shaped curve. Therefore, G_{tot} exhibits stable polarization at two minimum points. The point at which polarization can exist is the point where the derivative of G_{tot} with respect to P_s is zero, which can be expressed as

$$2(\alpha t_{f1} + xt_{f2})P_s + 4(\beta t_{f1} + yt_{f2})P_s^3 = V \text{ (S7)}$$

Differentiating G_{f1} with respect to P_s in Eq. (S3) and multiplying both sides by t_{f1}, and differentiating G_{f2} with respect to P_s in Eq. (S4) and multiplying both sides by t_{f2}, yields the following equations:

$$V_{f1} = (2\alpha P_s + 4\beta P_s^3)t_{f1} \text{ (S8)},\ V_{f2} = (2xP_s + 4yP_s^3)t_{f2} \text{ (S9)}$$

Equations (S8) and (S9) are shown in Figure S6(a) and S6(b), respectively. Combining Eq. (S7) and (S8) yields

$$V_{f1} = V - 2xt_{f2}P_s - 4yt_{f2}P_s^3 \quad \text{(S10)}$$

Equation (S10) is equivalent to substituting $V_{f2} = V - V_{f1}$ for V_{f2} in Eq. (S9). Therefore, the green curve in Figure S6c has a negative slope of the S-curve in Figure S6b, and the x-intercept represents V corresponding to the red square. Consequently, Figure S6d shows each curve at once as separately considering the right and left sides of Eq. (S10). Thus, it is critical to consider the intersections between the two curves in Figure S6d, which is the point at which polarization can exist stably.

L–K theory extension for MFIM capacitor

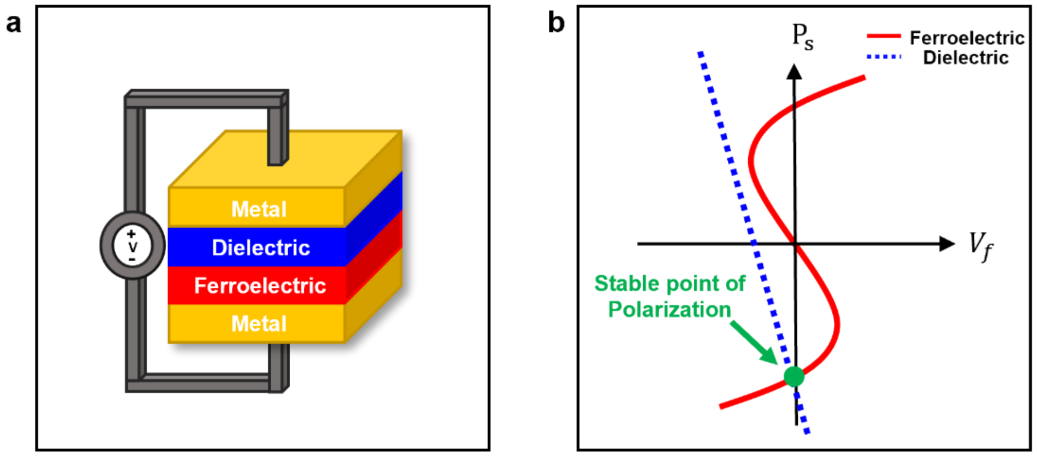

Figure S7. a) Schematic of metal/ferroelectric/insulator/metal (MFIM) capacitor. b) Stable point of polarization in MFIM capacitor. The red S-curve is for the ferroelectric, and the blue line is for the dielectric in the MFIM capacitor. The intersection between the red S-curve and blue line represents the stable point of polarization.

The Gibbs free energy of a dielectric with approximating D to P_s is given by

$$G_d = \frac{{P_s}^2}{2\varepsilon_0\varepsilon_d} - E_d P_s \quad \text{(S11)}$$

The shape of Eq. (S11) is parabolic, and if G_d is differentiated with respect to P_s, it becomes linear. This can be expressed as

$$E_d = \frac{t_d}{\varepsilon_0\varepsilon_d} P_s \quad \text{(S12)}$$

In Figure S7a, the total Gibbs free energy per area of the MFIM structure is given by

$$G_{tot} = G_f t_f + G_d t_d = \left(\alpha t_f + \frac{t_d}{2\varepsilon_0\varepsilon_d}\right) P_s^2 + \beta t_f P_s^4 - E_f t_f P_s - E_d t_d P_s \quad \text{(S13)}$$

G_f is the Gibbs free energy of the ferroelectric, (α, β) is the Landau coefficient of G_f, and G_d is the Gibbs free energy of the dielectric. t_d and t_f are the thicknesses of the dielectric and the ferroelectric, respectively. Equation (S13) can be converted to Eq. (S14) because ($V_f = E_f t_f$, $V_d = E_d t_d$), and the total V is the summation of V_f and V_d. Therefore, Eq. (S14) can be written as

$$G_{tot} = G_f t_f + G_d t_d = \left(\alpha t_f + \frac{t_d}{2\varepsilon_0\varepsilon_d}\right) P_s^2 + \beta t_f P_s^4 - V P_s \quad \text{(S14)}$$

If we find the point where the derivative of G_{tot} with respect to P_s is zero, it can be written as

$$2\left(\alpha t_f + \frac{t_d}{2\varepsilon_0\varepsilon_d}\right) P_s + 4\beta t_f P_s^3 = V \quad \text{(S15)}$$

From Eq. (S2), multiplying both sides t_f yields

$$V_f = 2\alpha t_f P_s + 4\beta t_f P_s^3 \quad \text{(S16)}$$

Combining Equations (S15) and (S16), it can be rewritten as

$$V_f = V - \frac{t_d}{2\varepsilon_0 \varepsilon_d} P_s \quad \text{(S17)}$$

Equation (S17) shows a linear curve with P_s and V_f on the axes. Equation (S16) shows an S-shaped curve with P_s and V_f on the axes. Consequently, if we consider the right and left sides of Eq. (S17) separately, it reveals that the linear curve from Eq. (S17) and the S-shaped curve from Eq. (S16) have an intersection, as shown in Figure S7b. The intersection represents a stable point of polarization.

Hysteresis formation behavior of MFIM capacitor

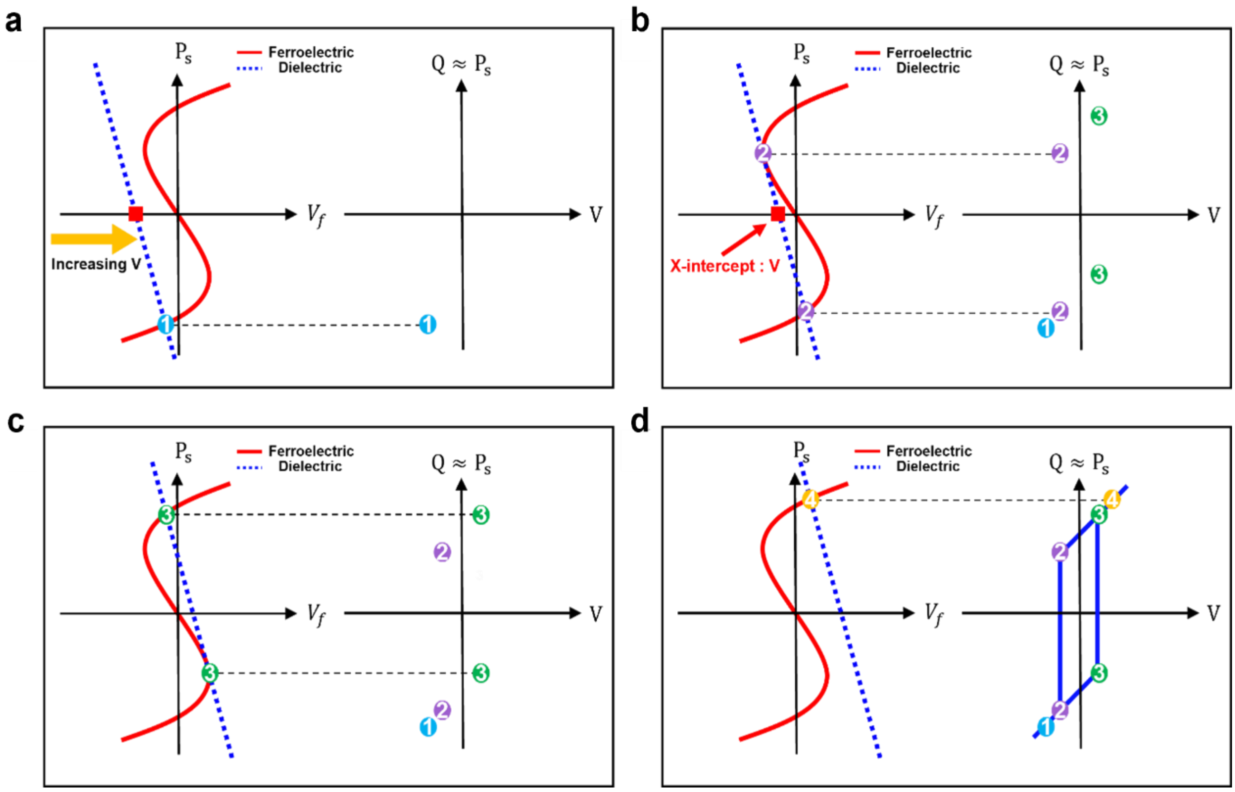

Figure S8. a) Blue line and the red S-curve intersect at one point. As the voltage increases, the blue line shifts to the left because *V* is x-intercept corresponding to the red square. (left). The intersection between blue line and red S-curve represents stable polarization. b), c) The blue line starts to have two intersections (left), which shows two stable polarization states in the system (right) with increasing voltage. d) Hysteresis window created in MFIM Capacitor.

Figure S8 shows the process of creating a hysteresis window with increasing voltage applied to the MFIM capacitor. On the left side of Figure S8a, the red square is the x-intercept, which represents V from Equation (S9). The right side of Figure S8a shows the quadrant with the approximation $Q \approx P_s$ and V as the axes. As the voltage applied to the MFIM capacitor increases, the blue line shifts to the red S-curve because V is the x-intercept of the blue line. The blue line intersects with the red S-curve at one point corresponding to 1, which can be a point in the quadrant on the right side of Figure S8a. Afterward, the blue line intersects with the red S-curve at two points corresponding to 2 and 3, as shown in Figure S8b and S8c. The two intersections between the blue line and red S-curve indicate that the MFIM capacitor can have two stable polarization states. In Figure S8d, the blue line and red S-curve intersect at

one point corresponding to 4 as the voltage further increases. On the right side of Figure S8d, if the points on the quadrant are connected, a hysteresis loop is formed. Therefore, to achieve a wider hysteresis window, the slopes and shapes of these two curves should be appropriately adjusted.

PFM phase width comparison between MFIM and MFFM

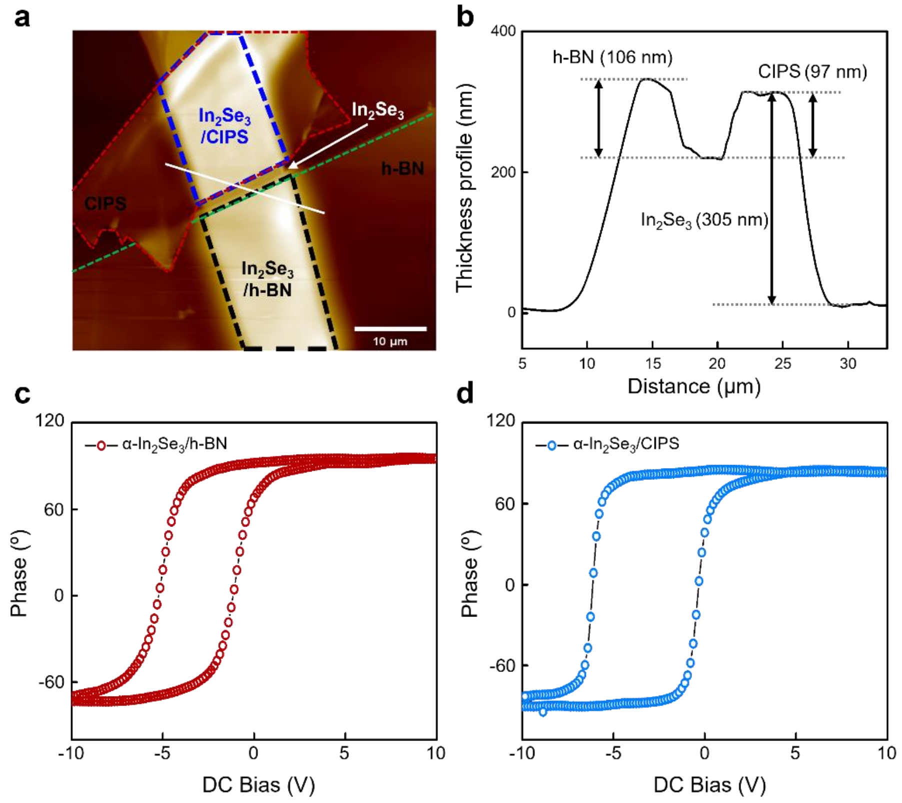

Figure S9. a) AFM topography image of α-In_2Se_3/h-BN and α-In_2Se_3/CIPS structures. The CIPS and h-BN are stacked on the same α-In_2Se_3. b) Thickness profile of α-In_2Se_3/h-BN and α-In_2Se_3/CIPS structures (along white line Figure S9a). c) PFM phase hysteresis loop for α-In_2Se_3/h-BN structure with hysteresis width of 4 V. d) PFM phase hysteresis loop for α-In_2Se_3/CIPS structure with hysteresis width of 6 V.

Figure S9a shows an AFM topography image of the α-In_2Se_3/h-BN and α-In_2Se_3/CIPS structures. CIPS (97 nm) and h-BN (106 nm) were stacked on the same α-In_2Se_3 (305 nm). The thickness profiles of the structures are shown in Figure S9b. Figure S9c–d show the PFM phase hysteresis loops for the α-In_2Se_3/h-BN and α-In_2Se_3/CIPS structures, respectively. The α-In_2Se_3/h-BN structure shows a hysteresis width of 4 V. In contrast, the α-In_2Se_3/CIPS structure shows a hysteresis width of 6 V, owing to the dipole coupling of each ferroelectric material. These results reveal that the ferroelectric/ferroelectric structure has wider hysteresis than the ferroelectric/dielectric structure when a dielectric and ferroelectric are stacked on the same ferroelectric with similar thicknesses. Consequently, the theoretical analysis discussed in Figure 3d is experimentally demonstrated in Figure S9.

Hysteresis characteristics in I_D–V_G curves of α-In_2Se_3/h-BN/CIPS Fe-FET

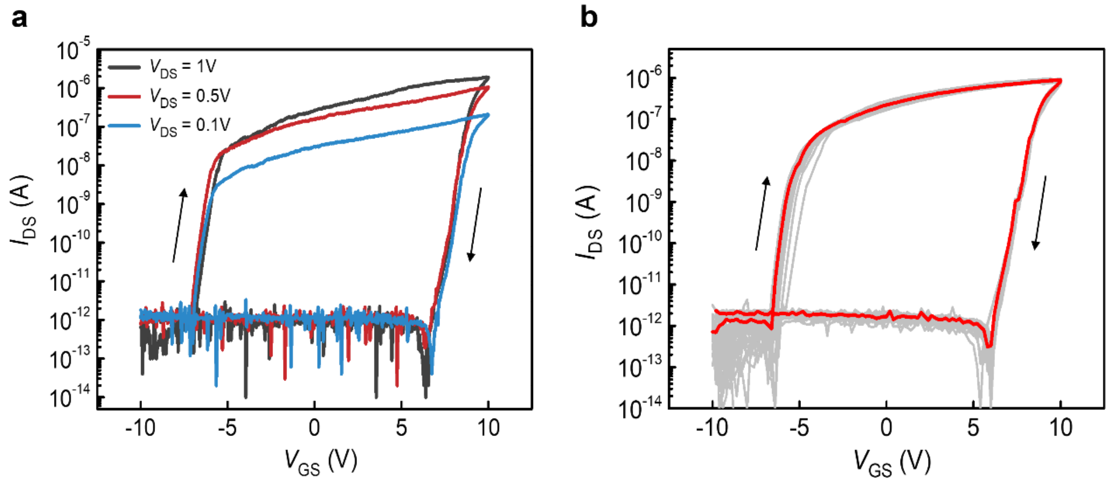

Figure S10. a) Transfer characteristics at V_{DS} = 1, 0.5, and 0.1 V. b) Transfer characteristics obtained with consecutive 50 cycles at V_{DS} = 1 V.

Figure S10a shows the transfer curves of the α-In_2Se_3/h-BN/CIPS Fe-FET according to the drain voltage. The gate voltage was swept from −10 to 10 V, and the channel current was measured at V_{DS} = 1 V (black), 0.5 V (red), 0.1 V (blue). The three transfer curves show n-type characteristics and clockwise hysteresis with a large memory window of 14.2 V in common. Figure S10b shows the reliability of the transfer curves. When the gate voltage was swept from −10 to 10 V for 50 cycles, the measured transfer curve exhibited stable behavior without significant degradation.

Data retention/Endurance characteristics of α-In_2Se_3/h-BN/CIPS Fe-FETs

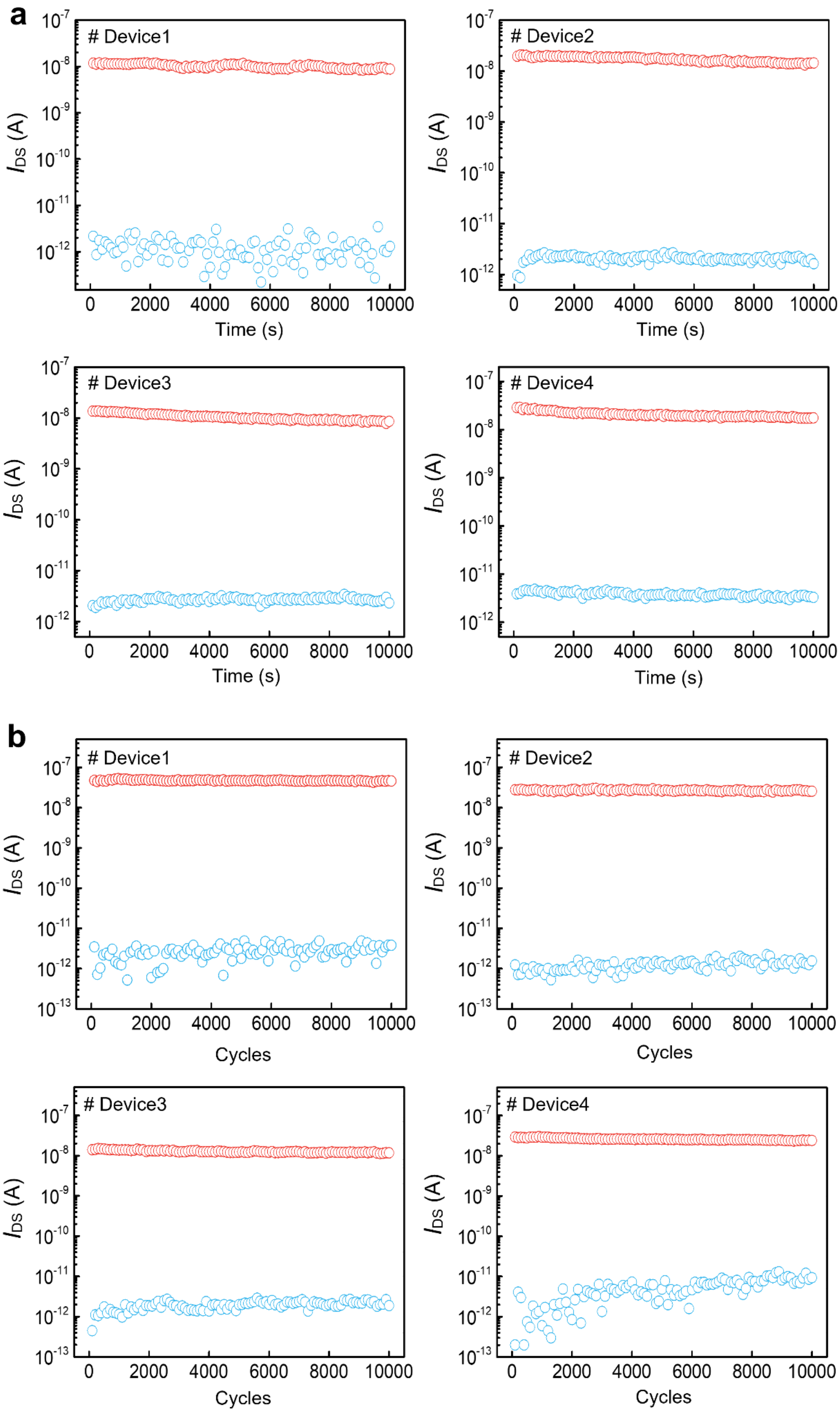

Figure S11. a) Raw data of the data retention characteristic shown in Figure 4c. After applying the programming/erasing pulses, the highly stable two states were maintained for longer than 10^4 s without significant degradation. b) Raw data of the endurance characteristics for four devices. The programming/erasing pulses with an amplitude of ±5 V and a duration of 1 s were repeatedly applied, resulting in stable two states for 10^4 cycles.

PPF characteristic of α-In_2Se_3/h-BN/CIPS ferroelectric synaptic device

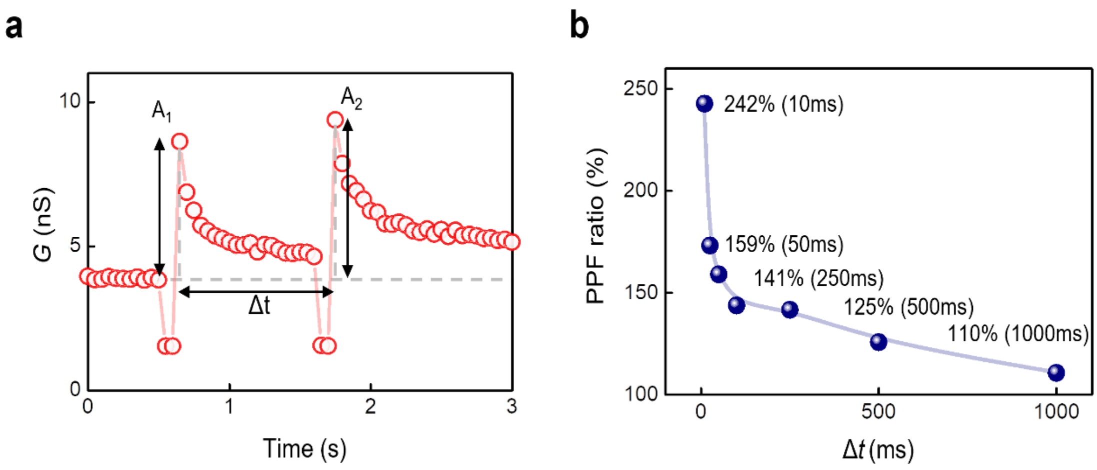

Figure S12. (a) Postsynaptic conductance response, where two electrical pulses with Δt were applied, and (b) PPF ratio as a function of Δt.

We investigated the PPF characteristics related to short-term plasticity, as shown in Figure S12. Two successive pulses (amplitude of −0.5 V, duration of 100 ms) with a time interval (Δt) were applied, and then we observed Δt-dependent facilitation behavior through the ratio of the first peak to the second peak in PSC (PPF ratio, A_2/A_1). The PPF ratio increased exponentially as Δt decreased (from 110% at Δt = 1000 ms to 242% at Δt = 10 ms), indicating that the PPF behavior of our synaptic device is similar to that of biological synapses.

Nonlinearity analysis of the LTP/LTD curves

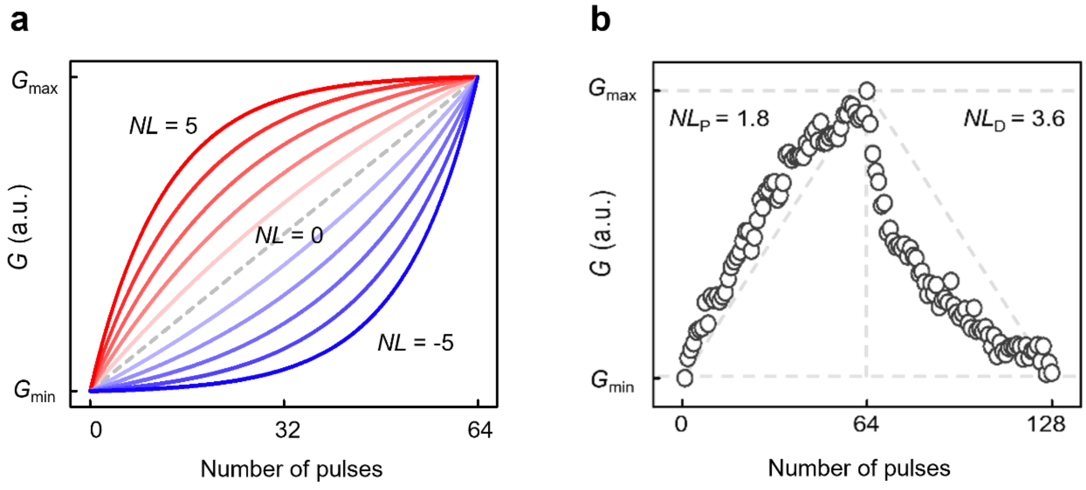

Figure S13. (a) LTP/LTD curves according to the nonlinearity (*NL*). (b) LTP/LTD curves of the α-In_2Se_3/h-BN/CIPS synaptic device, where the *NL* values are 1.8 and 3.6 for LTP/LTD curves, respectively.

The nonlinearity (*NL*) of the LTP/LTD curves was obtained using the following equations:

$$\boldsymbol{G_{\mathrm{LTP}} = B\left(1 - e^{\left(-\frac{P}{A_{\mathrm{P}}}\right)}\right) + G_{\mathrm{min}}}$$

$$\boldsymbol{G_{\mathrm{LTD}} = -B\left(1 - e^{\left(\frac{P-P_{\mathrm{max}}}{A_{\mathrm{D}}}\right)}\right) + G_{\mathrm{max}}}$$

$$\boldsymbol{B = (G_{\mathrm{max}} - G_{\mathrm{min}})/(1 - e^{\frac{-P_{\mathrm{max}}}{A_{\mathrm{P,D}}}})}$$

Here, G_{LTP} and G_{LTD} are the conductance values for the LTP and LTD curves, respectively. G_{max}, G_{min}, and *P* represent the maximum conductance, minimum conductance, and number of applied pulses, respectively. The parameter *A* is related to nonlinearity and *B* is a fitting constant used to normalize the conductance range. The *NL* values can be obtained using the *A* value from a table provided by DNN+ NeuroSim.[S5] Figure S13a shows the nonlinear behavior of the weight update according to the *NL* values. As shown in Figure S13b, the *NL* values obtained from the fitted LTP/LTD curves are 1.8 and 3.6, respectively.

LTP/LTD curves under various pulse conditions

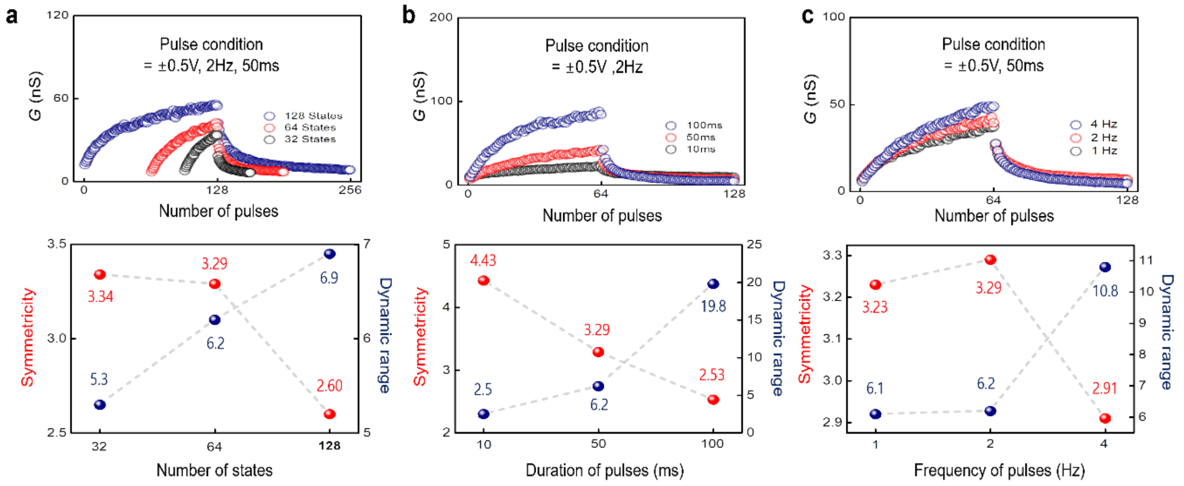

Figure S14. a) LTP/LTD curves according to the number of states (top) and extracted symmetricity and dynamic range values (bottom). b) LTP/LTD curves according to duration of pulses (top) and extracted symmetricity and dynamic range values (bottom). c) LTP/LTD curves with frequency dependency (top) and extracted symmetricity and dynamic range values (bottom).

As shown in Figure S14a–c, we investigated the LTP/LTD characteristics under various pulse conditions, including the number, duration, and frequency of pulses, and then extracted the DR (dynamic range, G_{max}/G_{min}) and symmetricity (for the symmetricity extraction method, see Figure S15). As the number of pulses increased from 32 to 128, the DR value increased from 5.3 to 6.9 while the symmetricity decreased from 3.34 to 2.60, as shown in Figure S14a. As the total electrical energy applied increases, the number of dipole switches increases, resulting in a large change in conductance. In addition to the number of pulses, the LTP/LTD curves according to the duration and frequency of the pulses showed similar results (Figure S14b–c). As longer or faster pulses were applied, the total electrical energy applied in the same period increased, resulting in a large change in conductance.

Symmetricity analysis of the LTP/LTD curve

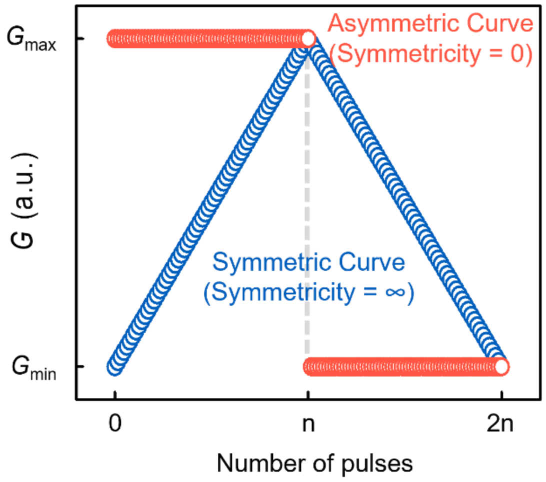

Figure S15. Symmetric (symmetricity = ∞) and asymmetric (symmetricity = 0) LTP/LTD curves.

Symmetricity is obtained with the following equation:

$$\text{Symmetricity} = \frac{1}{\text{Symmetric error}}$$

$$\text{Symmetric error} = \sum_{k=1}^{k=n} \frac{(G_\text{N}(k) - G_\text{N}(2n-k))^2}{n} = \sum_{k=1}^{k=n} \frac{((G(k) - G_\text{min}) - (G(2n-k) - G_\text{min}))^2}{n(G_\text{max} - G_\text{min})^2}$$

$$= \sum_{k=1}^{k=n} \frac{(G(k) - G(2n-k))^2}{n(G_\text{max} - G_\text{min})^2}, \text{ where } G_N(k) = \frac{G(k) - G_{min}}{G_\text{max} - G_\text{min}}.$$

Here, G_N, G_max, and G_min are the normalized, maximum, and minimum conductances, respectively.

Uniformity of LTP/LTD behaviors

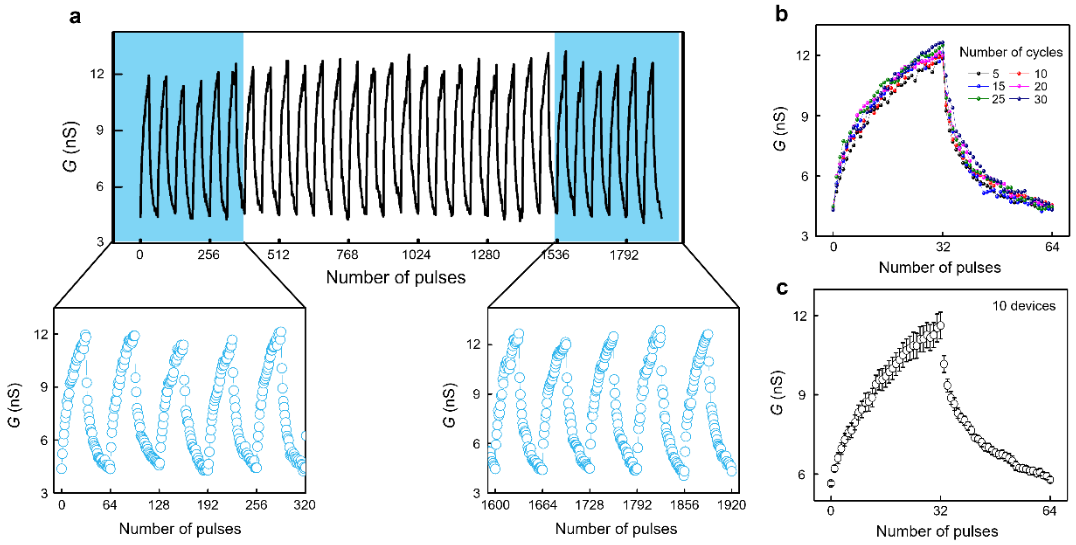

Figure S16. a) LTP/LTD curves of the α-In_2Se_3/h-BN/CIPS ferroelectric synaptic device for 30 cycles. The excitatory/inhibitory V_{wc} pulses with an amplitude of ±0.5 V, a duration of 10 ms and a frequency of 2Hz were applied for 30 cycles. b) Cycle-to-cycle variations of LTP/LTD curves for 30 cycles. Average cycle-to-cycle variation (σ/μ) is 2.89%. c) Device-to-Device variation for 10 devices. Average device-to-device variation (σ/μ) is 9.77%.

Convolutional neural network (CNN)

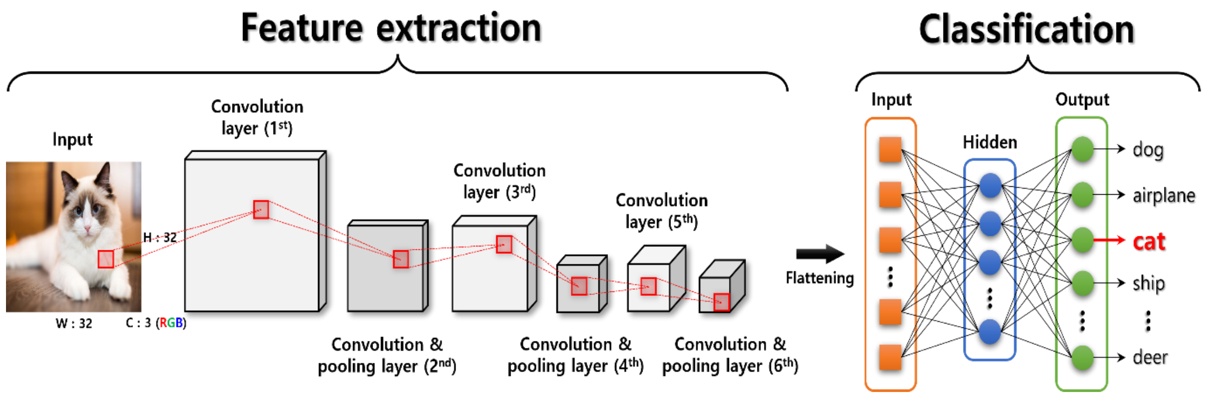

Figure S17. Schematic of CNN, which consists of six convolutional layers and two fully connected layers.

The layers of the CNN for extracting features from the images are shown on the left side of Figure S17. The input image data consist of 32×32 pixels and three RGB channels, and the value of the elements represents the voltage value. In the 1st layer of the CNN, for the convolution process, 3×3 kernel (synaptic weight) windows with a depth of 128 stride over the pixels of the images at intervals of 1 (i.e., input voltage value × kernel synaptic weight = current value). After this process, the size of the data decreased. Therefore, to prevent this, "zero padding." which adds zero values to the outside of the data, was performed. Through the convolution, feature maps were created, and the maps were activated by the ReLu activation function.[S6] In this process, the current values were converted into voltage values. The output values of the 1st layer were transferred to the 2nd layer, and higher-level features of the images were extracted using similar processes. In addition to these, "pooling," the process of reinforcing the extracted features, was performed. A 3×3 pooling window, which is the same size as the kernel windows used in the previous convolution process, was used, and the max pooling method that extracts the largest value among the element values within the window range was applied. Because of the pooling process, the size of the output data was smaller than that of the input data. The pooling processes were optionally applied to the 2nd, 4th, and 6th layers of this neural network.

These processes (convolution/convolution and max pooling) were repeated two more times, and the depth of the kernel windows was sequentially increased to 128, 256, and 512. As the data passed through the six layers for feature extraction, six convolution and three max pooling processes were performed. The voltage values of the 4×4×512 array were finally

output. Then, these values were flattened into a 1×8192 array to be transferred to the fully connected input layer.

The fully connected layers (FC layers) for image classification are shown on the right side of Figure S17. The FC layers consists of 8192 input neurons, 1024 hidden neurons (7th layer), and 10 output neurons (8th layer). We note that the input layer is not included when counting the number of layers. All neurons in each layer were fully connected to the neurons in the previous layer by synapses.

In the hidden layer (7th layer), the summation of weighted input values (**Σ**voltage value×synaptic weight) was activated by the ReLu function and converted to output voltage values. The output values of the 7th layer were passed to the input values of the 8th layer. A similar process occurred in the last output layer (8th layer), but the softmax function was used as the activation. After comparing the final output value of the 8th layer with the label value of the dataset, the synaptic weights for the convolutional kernels and FC were updated using a backpropagation algorithm (i.e., training tasks).[S7] Here, the synaptic weight (W) is defined as the difference between the conductance values of two ferroelectric synapses (W = G_P - G_D, where G_P and G_D denote the conductance values for potentiating and depressing, respectively). If G_P increases and G_D decreases simultaneously, the synaptic weight is potentiated (W↑ = G_P↑ - G_D↓). In contrast, synaptic weight was depressed when G_P decreases and G_D increases (W↓ = G_P↓ - G_D↑). These conductance values are related to nonlinearity *(NL)*, the number of applied pulses, and maximum/minimum conductance values, as shown in Figure S13. Consequently, the synaptic properties such as *NL*, multi-level states, symmetry/linearity of weight update, significantly influence the performance of neural networks.

Comparison of ferroelectric FETs reported thus far and their characteristics

	**Ref. 1**[S8]	**Ref. 2**[S9]	**Ref. 3**[S10]	**Ref. 4**[S11]	**Ref. 5**[S12]	**This Work**
**Type**	Fe-FET	Fe-FET	Fe-FET	FeS-FET	FeS-FET	vdWH Fe-FET
**Ferroelectric**	CIPS	PZT	P(VDF-TrFE)	α-In_2Se_3	α-In_2Se_3	α-In_2Se_3 CIPS
**Channel**	MoS_2	MoS_2	$MoSe_2$	α-In_2Se_3	α-In_2Se_3	α-In_2Se_3
**Gate insulator**	h-BN /CIPS	PZT	P(VDF-TrFE)	SiO_2	Al_2O_3 /h-BN	h-BN /CIPS
**On/OFF Ratio**	>10^7	>10^3	>10^5	>10^2	>10^4	>10^6
**Retention**	>10^4 s	>10^4 s	2×10^3 s	Not provided	500 s	>10^4 s
**Endurance**	300	100	>10^4	Not provided	500	>10^4
**Memory Window**	104V	6V	30V	50V	6V	14.47V
**Sweep Range**	±80V	±8V	±35V	±45V	±8V	±10V
**M.W./S.R.**	65%	38%	43%	56%	38%	72%

Table S1. Performance comparison of ferroelectric FET devices reported thus far and this work**.**

References

[S1] T. Liu, M. L. Lin, Y. C. Leng, X. Cong, X. Zhang, P. H. Tan, *J. Raman Spectrosc.* **2021**.

[S2] S. Miller, P. McWhorter, *J. Appl. Phys.* **1992**, 72, 5999.

[S3] R. Landauer, *Collect. Phenom.* **1976**, 2.

[S4] M. Hoffmann, S. Slesazeck, T. Mikolajick, *APL Mater.* **2021**, 9, 020902.

[S5] X. Peng, S. Huang, H. Jiang, A. Lu, S. Yu, *IEEE Trans. Comput.-Aided Des. Integr. Circuits Syst.* **2020**.

[S6] A. F. Agarap, arXiv: 1803.08375 (accessed: February 2019).

[S7] Y. LeCun, Y. Bengio, G. Hinton, *Nature* **2015**, *521*, 436.

[S8] W. Huang, F. Wang, L. Yin, R. Cheng, Z. Wang, M. G. Sendeku, J. Wang, N. Li, Y. Yao, J. He, Adv. Mater. **2020**, 32, 1908040.

[S9] X.-W. Zhang, D. Xie, J.-L. Xu, Y.-L. Sun, X. Li, C. Zhang, R.-X. Dai, Y.-F. Zhao, X.-M. Li, X. Li, *IEEE Electron Device Lett.* **2015**, 36, 784.

[S10] X. Wang, C. Liu, Y. Chen, G. Wu, X. Yan, H. Huang, P. Wang, B. Tian, Z. Hong, Y. Wang, *2D Mater.* **2017**, 4, 025036.

[S11] B. Tang, S. Hussain, R. Xu, Z. Cheng, J. Liao, Q. Chen, *ACS Appl. Mater. Interfaces* **2020**, 12, 24920.

[S12] S. Wang, L. Liu, L. Gan, H. Chen, X. Hou, Y. Ding, S. Ma, D. W. Zhang, P. Zhou, *Nat. Commun.* **2021**, 12, 1.
